# Introgression and rapid species turnover in sympatric damselflies

**DOI:** 10.1186/1471-2148-11-210

**Published:** 2011-07-18

**Authors:** Rosa A Sánchez-Guillén, Maren Wellenreuther, Adolfo Cordero-Rivera, Bengt Hansson

**Affiliations:** 1 Department of Ecology and Animal Biology, E. U. E. T. Forestry, Vigo University, 36005 Pontevedra, Spain; 2 Department of Biology, Lund University, SE-22362 Lund, Sweden

## Abstract

**Background:**

Studying contemporary hybridization increases our understanding of introgression, adaptation and, ultimately, speciation. The sister species *Ischnura elegans *and *I. graellsii *(Odonata: Coenagrionidae) are ecologically, morphologically and genetically similar and hybridize. Recently, *I. elegans *has colonized northern Spain, creating a broad sympatric region with *I. graellsii*. Here, we review the distribution of both species in Iberia and evaluate the degree of introgression of *I. graellsii *into *I. elegans *using six microsatellite markers (442 individuals from 26 populations) and five mitochondrial genes in sympatric and allopatric localities. Furthermore, we quantify the effect of hybridization on the frequencies of the genetically controlled colour polymorphism in females of both species.

**Results:**

In a principal component analysis of the microsatellite data, the first two principal components summarised almost half (41%) of the total genetic variation. The first axis revealed a clear separation of *I. graellsii *and *I*. *elegans *populations, while the second axis separated *I. elegans *populations. Admixture analyses showed extensive hybridization and introgression in *I. elegans *populations, consistent with *I. elegans *backcrosses and occasional F_1_-hybrids, suggesting hybridization is on-going. More specifically, approximately 58% of the 166 Spanish *I. elegans *individuals were assigned to the *I. elegans *backcross category, whereas not a single of those individuals was assigned to the backcross with *I. graellsii*. The mitochondrial genes held little genetic variation, and the most common haplotype was shared by the two species.

**Conclusions:**

The results suggest rapid species turnover in sympatric regions in favour of *I. elegans*, corroborating previous findings that *I. graellsii *suffers a mating disadvantage in sympatry with *I. elegans*. Examination of morph frequency dynamics indicates that hybridization is likely to have important implications for the maintenance of multiple female morphs, in particular during the initial period of hybridization.

## Background

Hybridization and introgression are increasingly recognized as important factors in the evolution of plants, animals [1,2] and prokaryotes [3], and can lead to the creation of novel genotypes and phenotypes. Thus, the study of contemporary hybridization between species and the extent of genomic introgression between them provides an excellent opportunity to examine evolutionary processes such as adaptation, gene flow and, ultimately, speciation [4-6]. Determining the degree of genetic exchange between species may be of particular interest when studying recently diverged species, since they typically show incomplete reproductive barriers.

Hybridization has an inherent spatial component, as the process requires direct contact between populations of the different species. For this reason, the spatial setting is a crucial determinant of hybridization, and, in turn, the specific conditions under which hybridization occurs can, sometimes, be inferred from the geographical distribution of hybrids. Studies of hybrid zones have indicated that natural hybridization is most likely to take place in intermediate habitats, which are often found at the ecological limits of the species' distributional ranges, and where both taxa are found in close proximity to each other [5]. When some of the interspecific matings lead to fertile first-generation (F_1_) hybrids, there is a possibility that these will backcross with at least one of the parental genotypes, with introgression as a consequence. If the resulting backcrossed individuals subsequently mate with the most similar parental genotype, novel genes and gene complexes can be particularly rapidly introduced into the new genetic background [7]. In some cases, stable and long-lasting hybrid zones are formed as a consequence of spatial range overlap between two species [8-10]. However, in the vast majority of cases, one of the two species, or possibly even the new hybrid cross, becomes more successful and displaces one or both of the original taxa [11].

In odonates (damselflies and dragonflies), a high level of hybridization between species is a rare phenomenon [12,13]. So far, only three molecular studies have investigated hybridization between closely related odonate species, namely between *Coenagrion puella *and *C. pulchellum *[14], *Mnais costalis *and *M. pruinosa *[15,16] and *Calopteryx splendens *and *C. virgo *[17]. All these studies failed, however, to detect extensive hybridization between the species. There was no evidence of hybridization between *Coenagrion puella *and *C. pulchellum *in any of the populations examined [14], i.e. no hybrids were found. In addition, between *Mnais costalis *and *M. pruinosa *only two F_1 _hybrid females were found among 900 individuals [15,16] and between *Calopteryx splendens *and *C. virgo *only seven hybrids out of 1600 putative hybrids were detected [17]. Despite the lack of evidence from molecular studies, observational and experimental studies have found evidence for putative intrageneric hybrids in some additional species [18]. For example, two of the best documented cases of hybridization among odonates are between *Ischnura gemina *and *I. denticollis *[19], and *I. elegans *and *I. graellsii *[20,21].

**Figure 1:**
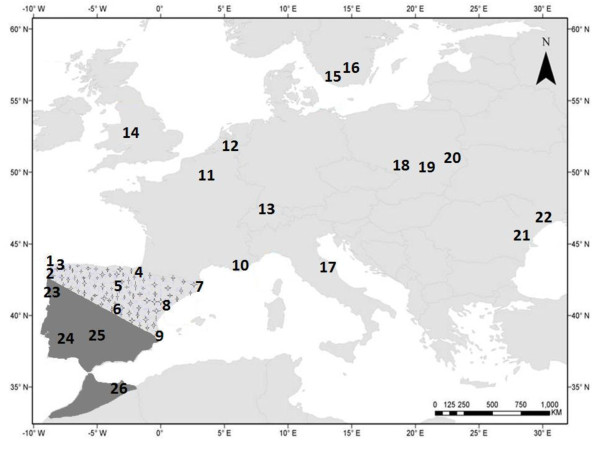
**Map showing spatial distribution of *I. elegans *and *I. graellsii *in Europe and northern Africa**. *I. elegans *(grey region) and *I. graellsii *(dark grey) in Europe and northern Africa, and the overlapping distribution of the two species in Spain (grey with stars). We sampled nine populations (166 samples) dominated by individuals that we phenotypically classified as *I. elegans *in three areas in Spain; north-west (1. Laxe, 2. Louro, 3. Doniños), north and central (4. Arreo, 5. Alfaro, 6. Baldajo) and east (Mediterranean coast: 7. Europa, 8. Amposta, 9. Marjal del Moro). Thirteen populations of allopatric *I. elegans *were sampled from Europe (10. Vigueirat, 11. Heuringhem, 12. Het Vinne, 13. Kaiserslautern, 14. Liverpool, 15. Värpinge, 16. Genarp, 17. Gran Sasso d'Italia, 18. Lublin-Zemborzyce, 19. Zwięczyca Reszów, 20. Breznica, 21. Suchoi Limon, 22. Enmakov Island; 220 samples). Finally, three populations of *I. graellsii *from the allopatric region in Iberia (23. Campus, 24. Ribeira de Cobres, 25. Córdoba) and one in northern Africa (26. Saïdia) (56 samples) were sampled.

The latter species pair consists of two closely related damselfly species that co-occur in southern Europe [18]. Specifically, *I. graellsii *is a widespread species on the Iberian Peninsula (see Figure 1), while *I. elegans *is a species with a more northern and easterly distribution, which has recently spread into new regions within the Iberian Peninsula [20,21]. For example, the first record of *I. elegans *in north-west Spain was made in 1984, whereas *I. graellsii *is known from this area since 1917 [22]. In addition, *I. elegans *is the dominant species in the coastal lagoons of Galicia (north-west Spain), a region where it was a very rare species less than 30 years ago. Furthermore, *I. elegans *is still rapidly expanding in the area, and in several coastal populations, that are dominated by *I. graellsii*, immigrant individuals of *I. elegans *are now starting to appear [21]. In a literature revision, Monetti *et al*. [20] have found that at least six Spanish localities that held both species simultaneously before the 1980's, had only *I. elegans *in 2002. Recent observations of *I. elegans *individuals in south Spain (personal observation), where only *I. graellsii *populations have been detected until now, and in western Spain, support that this species is gradually expanding its distribution in southern latitudes and western longitudes. *Ischnura elegans *has also expanded elsewhere; in the UK it has expanded its northern range by approximately 168 km in the last few decades, which is more than double than the average expansion distance of other odonates [23]. Likewise, Parmesan *et al*. [24] showed that 22 of 35 European butterfly species have shifted their ranges over the last century. The recent change in the climate has been suggested to drive changes in both phenology [25] and distribution [23] of odonates.

*Ischnura elegans *and *I. graellsii *are very similar ecologically and morphologically, but they can be unambiguously identified by the morphology of prothorax and anal appendages and by the comparatively small body and short wings of *I. graellsii *[20]. Furthermore, genetic analyses have shown that *I. elegans *and *I. graellsii *are very similar both at allozymes [26], and at the mitochondrial *Cytochrome b *and *Coenzyme II *genes [0.2% genetic distance, 21]. Previous work has documented that the two species hybridize in the laboratory [21], and hybrids (i.e. morphologically intermediate individuals) have been detected in one sympatric locality in north-western Spain [20].

Wirtz [27] reviewed the factors promoting unidirectional or reciprocal hybridization and proposed a hypothesis based on sexual selection to explain unidirectional hybridization. He proposed that hybridization is more likely between the female of the rare species and the male of the common species. However, when the rare species is also the bigger species, hybridization can be impeded by mechanical incompatibility [28], and the outcome degree and direction of hybridization is difficult to predict. Previous findings from the field (one population) [20] and laboratory [21] indicate that *I. graellsii *(the common and smaller species in Iberia) suffers a mating disadvantage in sympatry with *I. elegans *(the less abundant and larger species in Iberia). In particular, males of *I. elegans *readily mate with females of *I. graellsii *in the laboratory but males of *I. graellsii *are mechanically incapable of mating with *I. elegans *females [21]. The resulting F_1_-hybrid males can only mate with *I. graellsii *females, whereas the F_1_-hybrid females are mechanically incapable of mating with males of *I. graellsii *but can instead mate with *I. elegans *males [20]. Furthermore, F_1_-hybrids (males and females) show a similar degree of reduced viability and fertility [Sánchez-Guillén RA, Wellenreuther M and Cordero-Rivera A: Strong asymmetry in the relative strengths of prezygotic and postzygotic barriers between two damselfly sister species, submitted]. These conditions can be hypothesized to result in a directional bias in hybridization in favour of *I. elegans *that could explain the recently documented range expansion of *I. elegans *into areas that were previously only occupied by *I. graellsii *[20,21]. This agrees with the colonization pattern of *I. elegans *in the area. Under the described scenario of introgressive hybridization, we hypothesize extensive introgression of *I. graellsii *genes into the Spanish *I. elegans *populations. Furthermore, introgression and interspecific competition are probably contributing to the fast range expansion of *I. elegans *and contraction of *I. graellsii *in Iberia.

Interestingly, several *Ischnura *species, including *I. elegans *and *I. graellsii*, are characterized by a conspicuous colour polymorphism that is limited to females [21,29-31]. Females exhibit three colour morphs; one androchrome 1] and two gynochromes colour morphs (the green-brown *infuscans *and the orange-brown *infuscans-obsoleta *(*I. elegans*) or *aurantiaca *(*I. graellsii*)) [21,29]. The colour polymorphism is controlled by a simple genetic system consisting of one gene with three alleles that are in a dominance hierarchy [21]. How the colour polymorphism of *Ischnura *damselflies is maintained in space and time has been a much discussed subject. Indeed, hybridization was the first mechanism proposed for maintaining the colour polymorphism in this genus [32]. For example, hybridization was hypothesized to maintain contrasting androchrome frequencies in nearby populations of *I. damula *and *I. demorsa*, and *I. elegans *and *I. graellsii*, respectively [21,32]. Under the aforementioned scenario of extensive introgressive hybridization of *I. graellsii *genes into the Spanish *I. elegans *populations, and the hypothesised role of the hybridization for the temporal maintenance of contrasting androchrome frequencies; female morph frequencies of *I. elegans *in nearby populations to *I. graellsii *must be more similar to *I. graellsii *frequencies, due to the absorption of the typical morph frequencies, than in populations of *I. elegans *located far away of *I. graellsii *populations. In fact, Gosden & Svensson [33] proposed that the contrasting androchrome frequencies observed in the Spanish populations of *I. elegans *could be a by-product of the hybridization in combination with a founder effect.

The objectives of the present study were to examine the spatial distribution of *I. graellsii *and *I. elegans *in the Iberian Peninsula, evaluate the extent of introgression of *I. graellsii *genes into the recently established *I. elegans *populations in Spain, and to understand the role of the hybridization on the temporal maintenance of female colour morph frequencies in both species. Therefore, we conducted a detailed reconstruction of the distribution of the two species in Spain to quantify the overlap in space between the species. Furthermore, we examined the degree of introgression in nine *I. elegans *populations within the zone of overlap using both nuclear microsatellite markers [34] and mitochondrial genes [35]. Previous studies have shown that analyses based on differentiated markers between parental species can be used to efficiently and accurately describe admixture proportions, i.e. the degree of introgression, in F_1_-hybrids and backcrosses see [36-39]. Furthermore, studies have also shown the usefulness of mitochondrial DNA in the study of introgressive hybridization because of their maternal inheritance [40]. Specifically, with this method F_1_- and F_2_-hybrids may be assigned to a particular maternal species based on the mtDNA haplotype they carry. However, a prerequisite is that the species do not share haplotypes, which may happen because of hybridization and ancestral polymorphisms [41]. Finally, in order to evaluate if the previously observed uncharacteristic morph frequencies of *I. elegans *populations in north-western Spain, where frequencies are broadly different between populations [21], extend into the sympatric area (northern, central and eastern Spain), we combined morph frequency data from previous studies [42] with new data from this study, and discuss the proposed hypothesis about the role of hybridization in the temporal maintenance of the colour polymorphism.

## Results

### Spatial distribution of *I. graellsii *and *I. elegans *in the Iberian Peninsula

**Figure 2:**
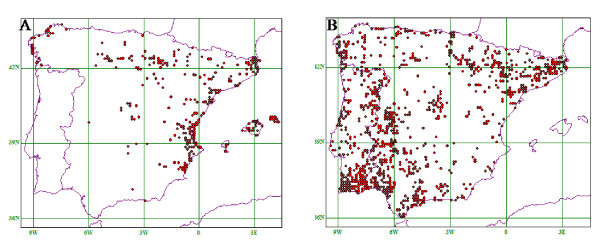
**Geographic distribution of *I. elegans *(A) and *I. graellsii *(B) in Spain**.

Using the available distribution data of *I. elegans *and *I. graellsii *in Spain, we constructed two geographic maps to show their overall distributional range (Figure 2A, *Ischnura elegans*; 2B, *I. graellsii*). Species overlap in north and central Spain (from west to east). In particular, *I. graellsii *is found all over the Iberian Peninsula, while *I. elegans *is very rare in southern Spain (Figure 2).

### Introgressive hybridization

All population groups (Spanish *I. elegans *populations, European *I. elegans *populations excluding Spain and *I. graellsii *populations) exhibited a high degree of molecular diversity (see Table 1). Estimates of observed and expected heterozygosity were similar and ranged from 0.56-0.70 and 0.61-0.76, respectively (Table 1). In the Spanish *I. elegans *populations, we detected a total of 87 alleles, 11 less than in the other European *I. elegans *populations. In contrast to *I. elegans*, we found somewhat fewer (66) alleles for *I. graellsii*, perhaps due to the fact that the microsatellites were specifically developed for *I. elegans *which could cause an ascertainment bias. Estimates of allelic richness were comparable between the Spanish and the other European *I. elegans *populations (6.54 and 6.03, respectively), and were considerably higher than in *I. graellsii *(3.41; Table 1).

**Table 1:** Basic population genetic data by regions and for each population:

Species	Populations	Region	N	H_O_	H_E_	Alleles	Richness
*I. elegans*	All Europe (except Spain)		220	0.701	0.764	98	6.031
*I. elegans*	Spain		166	0.705	0.767	87	6.538
*I. graellsii*	Iberia-Africa		56	0.577	0.682	64	3.409
*I. elegans*	Doniños	Spain	20	0.711	0.700	41	6.591
*I. elegans*	Laxe	Spain	14	0.715	0.805	32	6.146
*I. elegans*	Louro	Spain	15	0.712	0.729	32	5.792
*I. elegans*	Arreo	Spain	15	0.631	0.761	50	7.784
*I. elegans*	Baldajo	Spain	17	0.603	0.795	48	7.673
*I. elegans*	Alfaro	Spain	20	0.663	0.758	50	7.046
*I. elegans*	Europa	Spain	18	0.671	0.787	48	7.109
*I. elegans*	Amposta	Spain	20	0.691	0.770	51	7.156
*I. elegans*	Marjal del Moro	Spain	20	0.671	0.751	44	5.776
*I. elegans*	Lublin-Zemborzyce	East Europe	14	0.7505	0.797	60	8.081
*I. elegans*	Vigueirat	South France	16	0.733	0.804	42	6.252
*I. elegans*	Gran Sasso d'Italia	Central Italy	19	0.777	0.813	51	7.461
*I. elegans*	Liverpool	Great Britain	16	0.624	0.709	38	5.964
*I. elegans*	Heuringhem	North France	19	0.729	0.781	45	7.380
*I. elegans*	Kaiserslautern	South Germany	17	0.765	0.770	53	8.177
*I. elegans*	Het Vinne	Belgium	18	0.682	0.795	46	7.248
*I. elegans*	Höje Å	Sweden	20	0.653	0.717	43	7.010
*I. elegans*	Genarp	Sweden	20	0.680	0.753	44	7.203
*I. elegans*	Zwięczyca Reszów	East Europe	11	0.668	0.827	52	7.264
*I. elegans*	Breznica	East Europe	18	0.712	0.796	47	6.678
*I. elegans*	Suchoi Limon	East Europe	20	0.719	0.791	45	6.537
*I. elegans*	Enmakov Island	East Europe	15	0.713	0.766	49	6.811
*I. graellsii*	Campus	Spain	17	0.485	0.694	31	3.249
*I. graellsii*	Córdoba	Spain	20	0.647	0.653	36	3.466
*I. graellsii*	Ribeira de Cobres	Portugal	14	0.684	0.719	31	3.713
*I. graellsii*	Saïdia	North Africa	13	0.490	0.677	25	3.118

observed (H_O_) and expected heterozygosity (H_E_), number of alleles and allelic richness. N represents number of individuals genotyped per population.

Analyses of the overall genetic structure showed that *I. elegans *populations were significantly differentiated from one another in Europe outside Spain (F_ST _= 0.031, *P *< 0.0001) as well as within Spain (F_ST _= 0.049, *P *< 0.0001). Moreover, populations of *I. graellsii *were also significantly differentiated from one another (F_ST _= 0.029, *P *< 0.0001).

**Figure 3:**
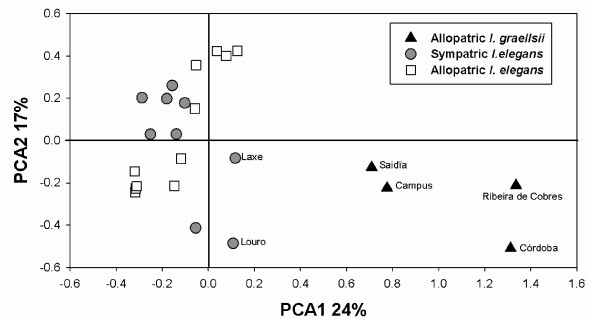
**Principal component analysis of allopatric *I. graellsii*, and allopatric and sympatric *I. elegans *populations**. The first (PCA 1) and second (PCA 2) axes represent the first two factorial components and sum up to 41% of explained variation.

Four of 23 principal component axes accounted for a significant amount of genetic variation among samples, as indicated by a screen plot. The first axis contained 24% (F_ST _= 0.024, *P *= 0.11), the second axis 17% (F_ST _= 0.017, *P *= 0.08), the third axis 14% (F_ST _= 0.014, *P *= 0.07) and the fourth axis 11% (F_ST _= 0.011, *P *= 0.05) of the total variation (Figure 3). The first two principal components thus summarise almost half (41%) of the total variation inherent in all *I. elegans *and *I. graellsii *populations. The scores for the first principal component axis revealed a clear separation of *I. graellsii *and *I*. *elegans *populations, suggesting that major population differences were predominantly caused by species rather than geographic areas *per se*. Major population differences (nested inside species) were revealed by the second PCA axis, where two of the Spanish *I. elegans *populations (Louro and Laxe; Table 2 and 3) were situated in the same quadrant that was occupied by all *I. graellsii *populations (Figure 3).

**Table 2:** Species, sampling locality, sample size for molecular analysis (N), ecology, sampling year, latitude and longitude.

Species	Locality	Ecology	N	Year	Latitude	Longitude
*I. graellsii*	Córdoba, southern Spain	allopatric	20	2008	37.8833	-4.7666
*I. graellsii*	Universitary-Campus, north-western Spain	allopatric	20	1999	42.171	-8.6778
*I. graellsii*	Saïdia, northern Africa	allopatric	20	2008	35.1217	-2.35
*I. graellsii*	Ribeira de Cobres, southern Portugal	allopatric	20	2005	37.496	-7.52
*Both species*	Arreo, north-central Spain	sympatric	20	2008	42.4775	-2.5787
*Both species*	Baldajo, central Spain	sympatric	20	2008	40.2426	-3.4206
*Both species*	Alfaro, north-central Spain	sympatric	20	2007	42.1908	-1.7423
*I. elegans*	Doniños, north-western Spain	sympatric	20	2007	43.2927	-8.1855
*I. elegans*	Laxe, north-western Spain	sympatric	20	2007	43.2125	-8.9554
*I. elegans*	Louro, north-western Spain	sympatric	20	2007	42.758	-9.0953
*I. elegans*	Europa, north-eastern Spain	sympatric	20	2008	42.2438	3.1028
*I. elegans*	Amposta, central-eastern Spain	sympatric	20	2008	40.2732	0.2156
*I. elegans*	Marjal del Moro, south-eastern Spain	sympatric	20	2008	39.0727	-0.3135
*I. elegans*	Liverpool, Great Britain	allopatric	20	2008	53.2439	-2.584
*I. elegans*	Värpinge, southern Sweden	allopatric	20	2005	55.7022	13.1437
*I. elegans*	Genarp, southern Sweden	allopatric	20	2005	55.6075	13.4042
*I. elegans*	Het Vinne, Belgium	allopatric	20	2007	50.833	5.117
*I. elegans*	Kaiserslautern, southern Germany	allopatric	20	2008	49.2641	7.4674
*I. elegans*	Heuringhem, northern France	allopatric	20	2008	50.4209	2.164
*I. elegans*	Vigueirat, southern France	allopatric	20	2008	43.5311	4.3012
*I. elegans*	Gran Sasso, central Italy	allopatric	20	2008	42.5015	13.4328
*I. elegans*	Lublin-Zemborzyce, Poland	allopatric	20	2007	51.15	22.34
*I. elegans*	Zwięczyca Reszów, Poland	allopatric	20	2007	50.0167	21.9167
*I. elegans*	Breznica, Poland	allopatric	20	2007	49.9696	19.6429
*I. elegans*	Suchoi Limon, Ukraine	allopatric	20	2006	46.03	30.047
*I. elegans*	Enmakov Island, Ukraine	allopatric	20	2006	45.435	29.525

**Table 3:** Number of *I.elegans*, *I. graellsii *and hybrid males examined in each putative introgressed populations.

Population	Region	Year	*I. elegans*	*I. graellsii*	*Hybrids*
Doniños	north-western Spain	2007	80	0	0
Laxe	north-western Spain	2007	206	0	0
Louro	north-western Spain	2007	136	0	0
Arreo	north-central Spain	2008	160	2	0
Alfaro	north-central Spain	2007	38	63	4
Baldajo	central Spain	2008	34	2	0
Europa	north-eastern Spain	2008	121	0	0
Amposta	central-eastern Spain	2008	30	0	0
Marjal del Moro	south-eastern Spain	2008	25	0	0

**Figure 4:**
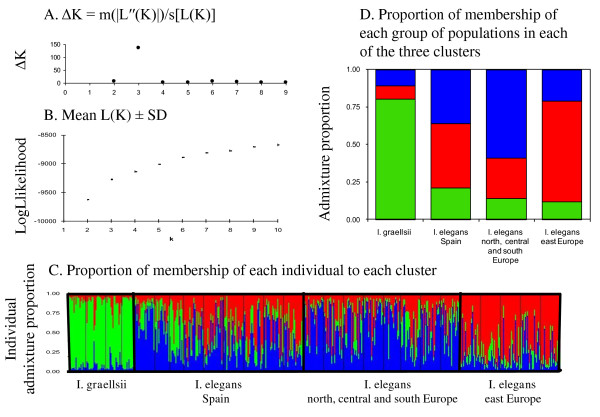
**Estimated population structure of *I. elegans *from Bayesian structure analyses using the program STRUCTURE**. **4.A**. *ΔK*-values for different *K*; suggesting *K *= 3 as the most likely structure (*ΔK *= m|L"(*K*)|/s[L(*K*)]; see [67]. **4.B**. Mean likelihood (± SD) of *K *for different numbers of clusters, *K*. **4.C**. Individual Bayesian assignment probabilities for *K *= 3 for 22 populations of *I. elegans *and 4 *I. graellsii *populations. Individuals are represented by thin vertical lines, which are partitioned into *K *shaded segments representing each individual's estimated membership fraction. The black lines separate sampling sites. **4.D**. The average Bayesian assignment probabilities for *K *= 3 for the four geographical areas; (1) eastern Europe, (2) northern, central and southern Europe, (3) Spanish *I. elegans *populations, and (4) *I. graellsii *populations.

Analyses of the population structure in STRUCTURE supported the molecular differentiation between the two *Ischnura *species, although the *ΔK*-method suggested three clusters as the most likely population structure (Figure 4). More specifically, the results showed that one of the genetic clusters highly corresponded to the *I. graellsii *group, while the other two genetic clusters were best represented by *I. elegans *genotypes (one mainly corresponding to northern, central and southern European populations, and one to populations in eastern Europe), with the Spanish populations showing an intermediate assignment to each of these three genetic clusters (Figure 4).

**Figure 5:**
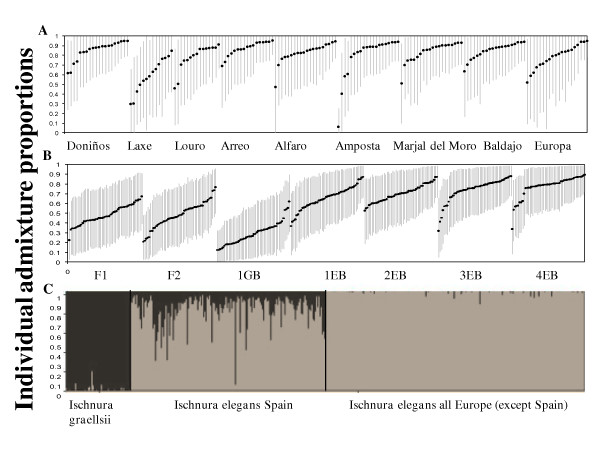
**Admixture analysis in STRUCTURE**. **A **and **B **panels show the admixture analysis in STRUCTURE of 166 genotypes of nine Spanish populations of *I. elegans *(upper panel) and for the artificial hybrids genotypes generated with the program HYBRID-LAB (mid panel). The estimated admixture proportion of each individual is represented by the mean assignment (± 90% credible intervals) to the *I. elegans *cluster. **C **panel shows admixture proportions of all 472 *I. elegans *and *I. graellsii *genotypes. Each individual is represented by a single vertical line broken into two segments which are proportional to the estimated membership in each of the two genetic clusters (Q_1 _for *I. graellsii *(black), and Q_2 _for *I. elegans *(grey)).

Based on these results, we included all populations of *I. graellsii *and *I. elegans *in the STRUCTURE runs (*K *= 2) to analyse the individual admixture proportions of *I. elegans *individuals in Spain (Table 4). These results showed that one genetic cluster clearly corresponded to *I. graellsii*, while the other cluster corresponded to *I. elegans *(European populations outside Spain; Table 4; Figure 5). The majority of individuals of the European *I. elegans *outside Spain (95%) and *I. graellsii *(88%) were assigned with a certainty of at least 90% to each of these clusters. However, only 27% of individuals of the Spanish *I. elegans *were strongly assigned to *I. elegans *(admixture proportions ≥ 90%) and one individual (from the sympatric population Alfaro) was assigned to *I. graellsii *(admixture proportion to *I. elegans *< 10%). The rest of the Spanish *I. elegans *individuals were intermediate between the two clusters (with a skew of admixture proportion towards *I. elegans*, see additional file 1), suggesting a significant degree of introgressed *I. graellsii *alleles (Table 4; Figure 5).

**Table 4:** Summary of the results from the Admixture models in STRUCTURE for *Ischnura *populations.

Admixture proportion to *I. elegans *cluster							
Region/Locality	N	Species	≥ 90	(89-68)	(67-21)	(20-11)	≤ 10
Iberia-Africa	56	*I. graellsii*	0	0	3	4	49
All Europe (except Spain)	220	*I. elegans*	209	11	0	0	0
Spain	166	*I. elegans*	45	97	23	0	1

*Spanish localities*							
Doniños	20	*I. elegans*	8	10	2	0	0
Laxe	14	*I. elegans*	0	5	9	0	0
Louro	15	*I. elegans*	1	12	2	0	0
Arreo	17	*I. elegans*	7	10	0	0	0
Baldajo	20	*I. elegans*	4	15	1	0	0
Alfaro	20	*I. elegans*	7	9	3	0	1
Europa	20	*I. elegans*	8	11	1	0	0
Amposta	20	*I. elegans*	6	13	1	0	0
Marjal del Moro	20	*I. elegans*	4	12	4	0	0

The admixture proportions of the artificial hybrids and backcrosses ranged between 11-89% (Table 5; Figure 5). The F_1 _and F_2 _showed admixture proportions to *I. elegans *between 67 and 21%; the 1 GB (first *I. graellsii *backcross) between 67 and 11%; and the four *I. elegans *backcrosses (1-4 EB) between 89 and 21% (Table 5; Figure 5). Based on these results, we defined three conservative assignment groups: backcrosses with *I. elegans *with admixture between 89-68%; backcrosses with *I. graellsii *ranging between 20-11%; and a mixed group of F_1_, F_2 _and backcrosses ranging between 67-21%. When the Spanish *I. elegans *individuals (N = 166) were allocated by their admixture proportions in one of these three groups, a total of 97 individuals (admixture proportions to *I. elegans *between 89-68%) were assigned to backcrosses with *I. elegans *(1-4 EB), whereas not a single individual was assigned to the backcross with *I. graellsii *(1 GB) (Table 5). Finally, the rest of the Spanish *I. elegans *(i.e. 23 individuals with admixture proportions to *I. elegans *between 67-21%), excluding the single individual from Alfaro that was assigned to *I. graellsii*, were assigned to the mixed-hybrid group, including F_1_, F_2 _and backcrosses with the parental species (Table 4).

**Table 5:** Summary of the results from the Admixture models in STRUCTURE for artificial hybrids and backcrosses:

Admixture proportion to *I. elegans *cluster						
Type of cross	N	≥ 90	(90-68)	(67-21)	(20-11)	≤ 10
F1	50	0	0	50	0	0
F2	50	0	0	50	0	0
1 GB	50	0	0	35	15	0
1 EB	50	0	24	26	0	0
2 EB	50	0	30	20	0	0
3 EB	50	0	40	10	0	0
4 EB	50	0	42	8	0	0

first-generation hybrid (F_1_; i.e. *I. graellsii *× *I. elegans*), second-generation hybrid (F_2_; i.e. F_1 _× F_1_), first backcross with *I. elegans *(1 EB; i.e. F_1 _× *I. elegans*), first backcross with *I. graellsii *(1 GB; F_1 _× *I. graellsii*), second backcross with *I. elegans *(2 EB; 1 EB × *I. elegans*), third backcross with *I. elegans *(3 EB; 2 EB × *I. elegans*), and forth backcross with *I. elegans *(4 EB; 3 EB × *I. elegans*).

### Low genetic variation and shared polymorphism at mitochondrial genes

The alignments for the *Cytochrome C Oxidase I *and *II *(COI-COII), *Cytochrome B *(CYTB), *12S rRNA *(12S) and *NADH Dehydrogenase 1 *(ND1) fragments included 591 bp, 673 bp, 457 bp, 370 bp and 591 bp, respectively (Table 6). All new sequences were deposited in GenBank (accession numbers: HQ834794-HQ834810). COI fragment showed no polymorphic sites, revealing a unique haplotype (H1) that was shared by the two species. COII showed one polymorphic site, revealing two haplotypes (haplotype diversity, H = 0.409 ± 0.133; nucleotide diversity, π = 0.00122). Haplotype H2, the most abundant haplotype, was shared by the species in both allopatric and sympatric regions, while haplotype H3 appeared only in the three samples of *I. graellsii *from one allopatric region (Morocco). CYTB fragment showed two polymorphic sites revealing three haplotypes (H = 0.163 ± 0.099; π = 0.00053). Each species showed one unique haplotype (H5 and H6), in a single sample from one allopatric region (Greece and Morocco), respectively, while the rest of the samples of both species shared a common haplotype (H4). The 12S fragment showed three polymorphic sites revealing four haplotypes (H = 0.087 ± 0.047; π = 0.00024). *Ischnura elegans *showed three unique haplotypes (H7, H8, H9), each of which appeared in a unique sample (two haplotypes in samples from allopatric regions and one in a sample from a sympatric region), while the rest of the samples of both species shared the common haplotype H10. The last fragment, ND1, showed one polymorphic site, revealing two haplotypes (H = 0.250 ± 0.180; π = 0.00042). The most abundant haplotype (H11) was shared by both species (from allopatric and sympatric regions), while the second haplotype (H12) appeared only in one sample of *I. graellsii *(from an allopatric population in Portugal).

**Table 6:** Number of individuals genotyped per gene (N), sequence length (bp), number of haplotypes (S), haplotype diversity (H) and nucleotide diversity (π) for each of the five analysed mitochondrial genes.

Mitochondrial gene			Genetic measures			
	N	bp	S (*I. elegans*)	S (*I. graellsii*)	H	π
COI	13	591	H1	H1	0.000	0.00000
COII	12	673	H2	H2, H3	0.409 ± 0.133	0.00122
CYTB	24	457	H4, H5	H5, H6	0.163 ± 0.099	0.00053
12S	68	370	H7, H8, H9, H10	H10	0.087 ± 0.047	0.00024
ND1	8	591	H11	H11, H12	0.250 ± 0.180	0.00042

The genes are *Cytochrome C Oxidase I *and *II *(COI-COII), *Cytochrome B *(CYTB), *12S rRNA *(12S) and *NADH Dehydrogenase 1 *(ND1). GenBank Accession numbers: HQ834794-HQ834810.

### Colour morph frequencies

All populations showed all three female morphs, with the exception of Saïdia in northern Africa (where the *aurantiaca *morph was missing) (Table 7). In populations dominated by *I. elegans *from north-central, central and eastern Spain, androchrome, *infuscans *and *infuscans-obsoleta *frequencies were highly variable between populations (range of androchrome frequencies: 3.3-42.9%; *infuscans*: 6.7-68.8%; *infuscans-obsoleta*: 11.8-76.7%) which is consistent with what has previously been observed for the *I. elegans *populations from north-western Spain [21]. In allopatric populations of *I. graellsii*, androchrome frequencies were always below 20% (3.0-18.8%); with the *infuscans *morph being the most abundant morph type (64.9-89.7%). The sympatric populations, O Vilar and Xuño, which have previously shown very high frequencies of androchromes for both species [21], also showed in our study high levels of androchrome frequencies (12.5-15.7% in *I. graellsii *and 40.0-47.6% in *I. elegans*). In addition, the sympatric populations Las Cañas and Alfaro from central Spain further showed high androchrome frequencies (13.0-17.1% in *I. graellsii *and 69.7-70.8% in *I. elegans*).

**Table 7:** Frequencies of female colour morphs of *Ischnura elegans *and *I. graellsii*.

Species	Ecology	Locality	Date	N	A	O	I	Source
*I. graellsii*	Allopatric	Saïdia, northern Africa	Jun-09	29	10.3	89.7	0	39
*I. graellsii*	Allopatric	Puente de los Arenales, southern Spain	Sep-08	33	3	78.8	18.2	39
*I. graellsii*	Allopatric	Doñana, southern Spain	Jun-03	77	10.4	76.6	13	21
*I. graellsii*	Allopatric	Granjuela, southern Spain	Sep-08	35	11.4	77.1	11.4	39
*I. graellsii*	Allopatric	Jaraiz de la Vera, central Spain	Jun-07	67	13.5	64.9	21.6	39
*I. graellsii*	Allopatric	Ribeira de Cobres, southern Portugal	Apr-2003	48	18.8	72.9	8.3	21
*I. graellsii*	Sympatric	Troi, northern Spain	Jul-08	54	14.8	72.2	13	39
*Sympatric-I. graellsii*	Sympatric	O Vilar, north-western Spain	May-Jul 2006	121	15.7	33.06	51.24	This study
*Sympatric-I. elegans*	Sympatric	O Vilar, north-western Spain	May-Jul 2007	21	47.62	47.62	4.76	This study
*Sympatric-I. graellsii*	Sympatric	Xuño, north-western Spain	Jun-Sept 2006	48	12.5	33.33	54.17	This study
*Sympatric-I. elegans*	Sympatric	Xuño, north-western Spain	Jun-Sept 2007	15	40	40	20	This study
*Sympatric-I. graellsii*	Sympatric	Las Cañas, north-central Spain	Jul-07	23	17.4	69.6	13	39
*Sympatric-I. elegans*	Sympatric	Las Cañas, north-central Spain	Aug-07	24	70.83	25	4.17	This study
*Sympatric-I. graellsii*	Sympatric	Alfaro, north-central Spain	Jul-07	23	13	60.9	26.1	39
*Sympatric-I. elegans*	Sympatric	Alfaro, north-central Spain	Aug-07	33	69.7	27.3	3	This study
*I. elegans*	Sympatric	Arreo, north Spain	Jul-08	30	6.3	68.8	25	39
*I. elegans*	Sympatric	Almoquera, central Spain	Aug-2008	28	42.9	42.9	14.3	39
*I. elegans*	Sympatric	Baldajo, central Spain	Aug-2008	34	29.4	58.8	11.8	39
*I. elegans*	Sympatric	Europa, north-eastern Spain	Jul-08	30	16.7	6.7	76.7	39
*I. elegans*	Sympatric	Amposta, north-eastern Spain	Jul-08	30	3.3	33.3	63.3	39
*I. elegans*	Sympatric	Barranco de Caixanet, south-eastern Spain	Sep-08	27	25.9	25.9	48.1	39
*I. elegans*	Sympatric	Marjal del Moro, south-eastern Spain	Sep-08	25	36	20	44	39

The number of mature female examined in each locality is indicated (N). A: *androchrome*, I: *infuscans *and O: *infuscans-obsoleta *(*I. elegans*) or *aurantiaca *(*I. graellsii*).

## Discussion

Hybridization and genomic introgression are repeatedly suggested to be important elements in evolutionary processes such as maintenance of genetic variation, adaptation and speciation [4-6]. Determining the degree of genetic exchange between recently diverged species may be of particular interest in this sense since they typically show incomplete reproductive barriers. Moreover, the pattern and extent of hybridization and introgression can have important conservation implications because it may lead to the replacement of one of the hybridizing taxa [10,43-45].

In the present study, we have revealed extensive hybridization and introgression in *I. elegans *populations in Iberia where it co-occurs sympatrically with its closely related sister species *I. graellsii*. Distribution maps for both species show an extended area of overlap in northern and central Spain. *Ischnura graellsii *is generally very abundant all along the Iberian Peninsula, while *I. elegans *has a patchy distribution. For example, *I. elegans *is very rare in southern Spain. In the sympatric regions, *I. elegans *is also less abundant than *I. graellsii *(with the exception of the area around Valencia) and is not present at all in some provinces (Figure 2). Consequently, *I*. *graellsii *occurs allopatrically in southern Iberia (Figure 2), whereas *I. elegans *is exclusively found in areas that are also occupied by *I. graellsii*. Our admixture analyses in STRUCTURE revealed clear evidence of past and present hybridization and introgression between *I. elegans *and *I. graellsii *over a large geographic area in northern Spain. The degree of introgression in the populations is consistent with *I. elegans *backcrosses and occasional F_1_-hybrids. Thus, hybridization between these two damselfly species is a relatively recent and widespread phenomenon. Interestingly, the studied populations have been going through a recent species turn-over and are now dominated by *I. elegans *individuals that appear to carry introgressed *I. graellsii *genetic material. These kinds of dramatic demographic and genetic effects have not previously been documented in odonates, although it is known from other taxa. For instance, over the past century, the blue-winged (*Vermivora pinus*) warbler has rapidly replaced the golden-winged warbler (*V. chrysoptera*) over an extensive part of their hybrid zone in eastern north America. Marker-based analyses show asymmetric and rapid introgression from blue-winged warbler into golden-winged warbler in some areas and bidirectional maternal gene flow in others [46,47]. Rapid introgression has also been detected in other taxa, e.g. in pocket gophers (*Geomys bursarius major *and *G. knoxjonesi*) [48].

### Extent of hybridization and direction of introgression

In vertebrates, hybridization is particularly common in fish, where several hundred interspecific and intergeneric crosses have been reported, and in birds, with roughly 10% of all species known to have bred in nature with another species e.g. [5,27,46-50]. In comparison, detailed genetic studies of hybridization and introgression, and thus the knowledge about these phenomena, are lacking for most odonates [12,13]. As mentioned above, three previous genetic studies failed in detecting extensive hybridization between different pairs of odonate species [14-17]. Our admixture analysis in STRUCTURE showed that the *I. elegans *populations Louro and Laxe in north-western Spain had the highest degree of introgression of *I. graellsii *alleles. This is not surprising, given that north-western Spain is the region where hybridization between *I. elegans *and *I. graellsii *was most recently detected [20]. In 1990, *I. elegans, I. graellsii *and hybrids were found in Foz (north-western Spain), which is geographically close to Louro and Laxe. However, after only ten years, the population was dominated by individuals that were phenotypically classified as *I. elegans*, although morphological intermediate and *I. graellsii *males were occasionally detected [21]. The population Laxe was visited for the first time in June 2001 (two visits) and only *I. elegans *were detected, although at a low densities (between 0.2 to 1.3 captured males/minute). At a visit in June 2002, *I. elegans *were found at a similar density (0.5 males/minute). Five years later (in 2007), we revisited the population and the density had reached the highest value in the region. A total of seven visits were conducted in 2007, between June and August, and the density ranged between 3.3 and 14.7 males/minute; and neither *I. graellsii *nor putative hybrids were detected. Nevertheless, the admixture analyses showed that none of the 14 examined individuals at Laxe could be genetically assigned to pure *I. elegans *status. Nine individuals were assigned to F_1_, F_2_, or backcrosses with *I. elegans *(admixture proportion between 67-21%), and five individuals were assigned to backcrosses with *I. elegans *(admixture proportion between 89-68%). In 1980, Louro was visited for first time by Ocharan [22] and mainly *I. graellsii *individuals were observed. However, Torralba and Ocharan [51] reviewed Ocharan's samples from Louro (sampled in 1980) and have now identified both species in the sample; nevertheless, *I. graellsii *species still remains the dominant species. However, on our first visit in 2000 and on subsequent visits, *I. elegans *individuals completely dominated in numbers (a couple of *I. graellsii *individuals were detected among hundreds of *I. elegans*). Only one out of the 15 Louro individuals that were molecularly examined in the present study was assigned to be pure *I. elegans*. In addition, the vast majority of the individuals (93%) showed an assignment proportion between 89-68%, suggesting that these individuals were backcrosses, and not F_1 _or F_2 _hybrids.

These results are corroborated by the PCA-analysis. Both Laxe and Louro were placed within the *I. graellsii *quadrant, indicating a significant degree of molecular similarity between these *I. elegans *populations and *I. graellsii*. Analyses of the remaining Spanish *I. elegans *populations showed that only 31% of the individuals were pure *I. elegans*, and that the remaining 69% showed admixture proportions expected for hybrids and backcrosses with *I. elegans*. In the Alfaro population in the north-central Spain where both species co-occur in equal numbers, one individual classified as *I. elegans *had a very high proportion of *I. graellsii *alleles. This either suggest that this individual was misclassified despite the fact that we only collected males to minimise misidentifications [20], or that it is a backcross that has inherited a very high proportion of *I. graellsii *alleles at the few markers analysed.

Our suggest that hybridization between *I. elegans *and *I. graellsii *is asymmetric and largely unidirectional. This corroborates previous findings in the field [20] and laboratory [21] showing that *I. graellsii *has a mating disadvantage in sympatry with *I. elegans*. Heterospecific matings, and matings between the F_1_-hybrids and the parental species, rarely take place with *I. elegans *females, but occur more frequently among *I. graellsii *females, *I. elegans *males and hybrids [Sánchez-Guillén RA, Wellenreuther M and Cordero-Rivera A: Strong asymmetry in the relative strengths of prezygotic and postzygotic barriers between two damselfly sister species, submitted]. The reason for the almost complete lack of hybridization between *I. graellsii *males and *I. elegans *females is that males cannot grasp the female by their protothorax [20], a mechanical handicap that appears to be a very efficient prezygotic isolation mechanism. Previous work on plants [52,53] and animals [27] has suggested that unidirectional hybridization usually occurs between the females of the rare species and the males of a common species, but not *vice versa*. Wirtz [27] reviewed the factors promoting unidirectional or reciprocal hybridization and proposed a sexual selection hypothesis for unidirectional hybridization based on the fact that females normally invest more in offspring and, therefore, are more discriminating than males. When heterospecific males are less abundant than conspecific males, females rarely mate with heterospecific males. Consequently, under such a condition, the rare species is usually the maternal parent of the hybrids. However, this is not the case in our study where the more abundant species is initially *I. graellsii*. *Ischnura elegans*, on the other hand, appears to be the intruding species and is hence initially the rare species, which has been expanding its range in Spain and is now displacing *I. graellsii *from some populations and regions. Thus, the direction of hybridization between these two *Ischnura *species is opposite that expected based on the rare female hybridization hypothesis just outlined e.g. [27], but follows the prediction proposed by Grant and Grant [28], namely that when the rare species is also the bigger species (in our study *I. elegans*), hybridization can be impeded by mechanical incompatibility. Laboratory tests have detected that *I. elegans *females and *I. graellsii *males are mechanically impaired to form a tandem, preventing over 93% of all matings, while only 13% of the matings between *I. graellsii *females and *I. elegans *males are mechanically prevented [Sánchez-Guillén RA, Wellenreuther M and Cordero-Rivera A: Strong asymmetry in the relative strengths of prezygotic and postzygotic barriers between two damselfly sister species, submitted].

### The role of hybridization in the maintenance of colour polymorphisms

Johnson [33] proposed that the colour polymorphisms in odonates could be maintained due to hybridization between closely related species and balanced by differential predation pressure. According to this hypothesis, androchrome females benefit from avoiding matings with heterospecific males, while gynochromes females are involved in heterospecific matings (usually sterile); the relative fitness of the colour morphs is balanced by a higher probability of predation of the androchrome females. In a recent study on *I. elegans *and *I. graellsii *[21], a role of hybridization for the temporal maintenance of contrasting androchrome frequencies in nearby populations in north-western Spain was suggested. The relatively low androchrome frequencies in *I. elegans *populations located close to *I. graellsii *populations, and the relatively high androchrome frequencies in *I. graellsii *populations adjacent to *I. elegans *populations, were hypothesized to be caused by the absorption of the, in other circumstances, typical morph frequency [21]. *Ischnura elegans *data from other sympatric regions in the Iberian Peninsula now shows that the substantial variation in female morph frequencies between different populations in north-western Spain is not unique to this region; also in other parts of Iberia morph frequencies are drastically variable between populations (androchrome: 3.3-70.8%; *infuscans*: 6.7-72.9%; *infuscans-obsoleta*: 3.0-76.7%). Furthermore, other recent studies have revealed that disparate morph frequencies are not restricted to Iberian populations [53,54], although androchrome frequencies are typically more abundant in northern latitudes and show less variation between populations than in Iberia [with the exception of Iberia, 53]. In our study, the highest frequencies of androchromes were found in the four populations with both species present (Xuño, O Vilar, Alfaro and Las Cañas; Table 7); in *I. graellsii *androchrome frequencies ranged between 12.5 and 17.4% and in *I. elegans *between 40.0 and 70.8%. Nevertheless, the *I. graellsii *population Ribeira de Cobres had androchrome frequencies of 18.8%, for this species an atypically high level. However, this comparatively high androchrome frequency cannot be explained by the influence of nearby *I. elegans *populations, because this population is located in the allopatric region (southern Portugal). Furthermore, in *I. elegans *the lowest androchrome frequencies were found in north-central Spain (population named Arreo) where the nearest *I. graellsii *population (named Troi) had higher androchrome frequencies (14.8%) than the *I. elegans *population (3.3%), and in a coastal population near the Mediterranean coast (eastern Spain) (Amposta; 3.3% androchromes), but here we lack data of *I. graellsii *morph frequencies in nearby populations.

In conclusion, hybridization is likely to have important implications for the maintenance of multiple female morphs, but only during the short period when two species with contrasting morph frequencies start to hybridize.

The evolution, maintenance and adaptive function of genetic colour polymorphism have received very much attention in a broad range of organisms, e.g. in birds [55], reptiles [56] and insects [57]. However, the role of hybridization in this context has received little attention, which may simply reflect the fact that only a few suitable study systems are available to study; one being the presently described *Ischnura *damselfly complex in Spain. Another case is the hybrid zone of the land snails *Mandarina mandarina *and *M. chichijimana *in the oceanic Bonin Islands [58]. In that system, the variability of the colour polymorphism in the hybrid population was substantially higher than that in the pure populations, suggesting that morphological variation was maintained by hybridization [58]. These results highlight the importance of hybridization as a source of morphological variation, diversity and evolutionary novelty.

## Conclusions

When hybrids are fertile and backcross with one of the parental species, hybridization will inevitably result in introgression, thereby increasing the genetic variability of the introgressed species [59]. Even at low levels, introgression of novel genetic material could be an important factor as a source of new genetic and phenotypic variability, and subsequent evolution. Thus a certain degree of hybridization could create favourable conditions for new adaptations [4-6]. In contrast, extensive hybridization and introgression can, as mentioned above, sometimes have important conservation implications when leading to the replacement of one of the hybridizing taxa [10,42-44]. We have documented a unique case of hybridization and unidirectional introgression in polymorphic *Ischnura *damselflies, where hybridization is likely to having important implications on the temporal maintenance of multiple female morphs. The potential adaptive significance of introgression in the system, and whether this *per se *has contributed to the rapid species turn-over in sympatric populations in the region, remains to be evaluated.

## Methods

### Spatial distribution of *I. graellsii *and *I. elegans *in the Iberian Peninsula

We conducted a revision of the distribution data of the two species along the Iberian Peninsula from 1866-2008 using data from Baixeras *et al*. [60], Jödicke [61] and Ocharan [22,62], in the region around la Rioja using data from Tomás Latasa (personal communication) and along the Iberia using data from Jean Pierre Boudot (personal communication). Using DMAP (Distribution mapping software, Version 7.0) we constructed two geographic maps showing the distribution of both species in Iberia (Figure 2).

### Study populations and sample collection

Samples of *I. elegans *and *I. graellsii *were collected from 26 populations from Europe and northern Africa (see Table 2 for details of sampling locations). In particular, all European populations, except the populations from Spain, were classified as allopatric *I. elegans *populations and included a total of 220 individuals from 13 *I. elegans *populations. In depth sampling of both *I. elegans *and *I. graellsii *was carried out in the sympatric region in Spain; in the north-western corner, the central parts and along the east coast (Figure 1). In these areas, nine sympatric populations (166 individuals) were sampled, and these were, with the exception of Alfaro, dominated by individuals that were phenotypically classified as *I. elegans *(see Table 3 for details of species proportions in the sampled populations). In addition, four allopatric *I. graellsii *populations (56 *I. graellsii *individuals) from the north, central and south Iberia, and northern Africa were sampled.

At each allopatric and sympatric *I. elegans *population (see Table 2), and at the four allopatric *I. graellsii *populations, a minimum of 20 adult males were collected during the flight season between 1999-2008 using hand nets. Captured individuals were stored in 100% ethanol until DNA extraction. Only males were sampled because the identification of male *I. elegans*, *I. graellsii *and hybrids is more reliable than that of females. Note that the few individuals classified as *I. graellsii *and hybrids in the Spanish *I. elegans *populations, mainly found in Alfaro in north-central Spain (Table 3), were not included in the genetic analyses because the aim of this study was to test for introgression in *I. elegans *populations.

### DNA extraction and microsatellite genotyping

To extract DNA from the samples, the head of each damselfly was removed, dried and then homogenized using TissueLyser (Qiagen). DNA was extracted by proteinase K digestion followed by a standard phenol/chloroform-isoamylalcohol extraction [63]. The purified DNA was re-suspended in 50-100 μl of sterile water. The genotypes of all damselflies were assayed at six microsatellite loci previously isolated for this species [I-002, I-015, I-041, I-053, I-095, I-134, for details see 33]. These loci did not deviate statistically from Hardy-Weinberg expectations and linkage equilibrium, and showed no evidence for presence of common null-alleles (using Micro-Checker; [64], within populations of both species [34]. One primer of each pair was 5'-labelled with 6-FAM, HEX or NED florescent dyes. Polymerase chain reactions (PCRs) were carried out in 10 μL volumes on a GeneAmp PCR System 9700 (Applied Biosystems) and contained 4 pmol of each primer, 15 nmol MgCl_2_, 1.25 nmol dNTP, 0.5 U Ampli-taq polymerase and 10-20 ng template. The conditions were: denaturation step of 94°C for 2 minutes, then 35 cycles at 94°C for 30 s, touch-down from 62-58°C for 30 s, 72°C for 30 s followed by 72°C for 10 minutes. Multiplex primer reactions were performed for combinations of primers with matching annealing temperatures but differing size ranges and dye labels, then mixed with a labelled size standard and electrophoresis was conducted on an ABI PRISM 3730 Genetic Analyzer (Applied Biosystems). GeneMapper 3.0 (Applied Biosystems) was used for fragment size determination and allelic designations.

### Microsatellite DNA Analyses

The program FSTAT [65] was used to calculate several basic population genetic measures, namely, the expected heterozygosity (H_E_), observed heterozygosity (H_o_), number of alleles, and the allelic richness for each population. These aforementioned measures as well as the genetic differentiation between populations (F_ST_) were also calculated for three regions, namely the Spanish populations of *I. elegans*, the European populations of *I. elegans *(excluding Spain), and the entire sample of *I. graellsii*.

Principal component analysis (PCA) was used to reduce the variation in the multivariate data set (consisting of 117 alleles at six loci) to two linear combinations. The analysis was done using PCA-GEN [66]. The significance of each principal component was assessed from 5000 randomisations of genotypes. The allocation of each species across the two principal components provides a quantitative measure of the degree of the genetic dissimilarity among the populations/species [18].

The Bayesian statistical framework provided by the program STRUCTURE [version 2.2.3, 67] was used to understand the genetic structure among populations and to determine which individuals from the allopatric and sympatric populations of *I. elegans *and *I. graellsii *can be classified to a high degree as pure species. STRUCTURE applies a Bayesian Markov chain Monte Carlo (MCMC) approach that uses model-based clustering to partition individuals into groups. The model accounts for the presence of Hardy-Weinberg and linkage disequilibrium by introducing group structure and attempts to find groupings that (as far as possible) are in equilibrium [68]. We applied the 'admixture model' with 'correlated allele frequencies' for more details [69]. For the model, a 'burn-in' period of 20,000 MCMC replicates and a sampling period of 100,000 replicates were used. We performed runs for a number of genetic clusters (*K*), ranging from one to ten; and for each *K*, 20 iterations were run. In this way, multiple posterior probability values (log likelihood (lnL) values) for each *K *were generated, and the most likely *K *was evaluated by the *ΔK*-method following Evanno *et al*. [66].

Admixture analyses in STRUCTURE were also used to assign all individuals of the Spanish *Ischnura *populations into each of two genetic clusters, one representing *I. graellsii *genotypes and one *I. elegans*. We used the 'prior population information' option in the models to (i) facilitate the clustering process of the reference individuals (i.e. pure *I. elegans *from central and eastern Europe, and *I. graellsii*, respectively), and (ii) to calculate the admixture proportions (and ± 90% credible regions) of each individual in the Spanish *I. elegans *populations. This approach was hence used to measure of the degree of introgression of *I. graellsii *genetic material into the genome of *I. elegans *in Spain. The model was run for *K *= 2, where one cluster corresponded to *I. graellsii *and the other to *I. elegans*. We used the 'population flag' option to exclude Spanish *I. elegans *as reference individuals, which implied that the clustering process was based on only *I. graellsii *samples and *I. elegans *samples collected outside of Spain. The model was run for 100,000 MCMC replicates, after an initial burn-in period of 20,000 replicates, using the admixture model and correlated frequencies for five iterations [32-34,63]. To generate simulated genotypes of hybrids and backcrosses, we applied the program HYBRID-LAB [70] using the genotypes of 66 individuals of *I. graellsii *and 240 genotypes of *I. elegans *collected outside of Spain as initial genotypes. We generated 50 genotypes of each of the following crosses: first-generation hybrid (F_1_; i.e. *I. graellsii *× *I. elegans*), second-generation hybrid (F_2_; i.e. F_1 _× F_1_), first backcross with *I. elegans *(1 EB; i.e. F_1 _× *I. elegans*), first backcross with *I. graellsii *(1 GB; F_1 _× *I. graellsii*), second backcross with *I. elegans *(2 EB; 1 EB × *I. elegans*), third backcross with *I. elegans *(3 EB; 2 EB × *I. elegans*), and forth backcross with *I. elegans *(4 EB; 3 EB × *I. elegans*). We then evaluated the admixture proportions (± 90% credible intervals) of these artificial crosses with STRUCTURE in the same way as done for the *I. elegans *samples from Spain (above). To determine the level of introgression of *I. graellsii *into the Spanish *I. elegans *populations, the individual admixture proportions of the *I. elegans *samples from Spain were compared to the admixture proportion for the artificial hybrids and backcrosses.

### Mitochondrial sequencing

Three to six damselflies from sympatric and allopatric localities were amplified by polymerase chain reaction (PCR) for part of the mitochondrial *Cytochrome C Oxidase I *and *II *genes (COI-COII), part of the mitochondrial *Cytochrome B *(CYTB) gene and part of the mitochondrial *12S rRNA *(12S) and *NADH Dehydrogenase 1 *(ND1). The amplification was done using universal primers: 591 bp of the COI with the primers COI-H (5'-TCAGGGTGACCAAAAAATCA-3') and COI-L (5'-GGTCAACAAATC ATAAAGATATTGG-3') (Marina Magaña Ramos, personal communication), 673 pb of the COII with the primers TL2-J-3037 (5'- ATGGCAGATTAGTGCAATGG-3') and C2-N-3494 (5'-GGTAAAACTACTCGATTATCAAC-3') and C2-J-3400 (5'-ATTGGACATCAATGATATTGA-3') and TK-N-3785 (5'-GTTTAAGAGACCAGTACTTG-3') [34], 457 pb of the CYTB with the primers CB-J-10933 (5'-TATGTACTACCATGAGGACAAATATC-3') and TS1-N-11683 (5'-TATTTCTTTATTATGTTTTCAAAAC-3') [34], 370 bp of the 12S with the primers SR-J-14233 (5'-AAGAGCGACGGGCGATGTGT-3') and SR-N-14588 (5'-AAACTAGGATTAGATACCCTATTAT-3') [30], and 591 bp of the ND1 gene with CB-J-11545 (5'ACATGAATTGGAGCTCGACCAGT-3') and N1-N-12051 (5'-GATTTTGCTGAAGGTGAATCAGA-3') [34]. DNA amplification was done in a total reaction volume of 20 μl. The amplification conditions were as follows: 50 ng of DNA (1 μL), 1 unit (0.2 μL) of Taq DNA polymerase (Ecogen), 2 μL 10x of reaction buffer (Ecogen), 0.5 μL of MgCl_2 _(50 mM) (Ecogen), 0.5 μL of dNTPs Mix Sigma (200 μM), and 1 μL of each primer (10 pmol). All PCR reactions were completed in a "GeneAmp PCR system 2700" thermocycler (Applied Biosystems). The PCR program had an initial cycle of 95°C for 3 min, the annealing temperature for 1 min, and an elongation period at 72°C for 45 s, followed by 34 cycles at 95°C for 30 s, with annealing for 45 s, and an elongation phase at 72°C for 45 s, and a final extension phase at 72°C for 10 min. PCR products were sent to an external sequencing service (University of Valencia) where bidirectional sequencing reactions were conducted using Bigdye™terminator cycle sequencing kit (Applied Biosystems) using automatic sequencer ABI3100 (Applied Biosystems). Forward and reverse sequences were edited in Codon Code Aligned (CodonCode, Dedham, MA, USA) and consensus sequences were aligned with Clustal X [71] implemented in MEGA v. 4.0 [72,73]. Variable positions were revised by eye, and only high quality sequences were considered.

### Mitochondrial data analyses

Samples of both species were pooled in two groups, depending on the sympatric or allopatric origin. For each gene, genetic diversity was assessed in terms of number of haplotypes (S), and haplotype diversity (H) and nucleotide diversity (π) according to Nei [74] with DNASP V4.10 [74]. Further analyses could not be done because overall low genetic diversity and shared haplotype frequencies.

### Colour morph frequencies

We reviewed female morph frequencies from the literature in seven *I. graellsii *populations from Iberia and northern Africa and seven *I. elegans *populations from Iberia [41]. In addition, we estimated female colour morph frequencies in four sympatric populations; Xuño and O Vilar from north-western Spain, and Alfaro and Las Cañas from north-central Spain. Populations were visited between June and September during sunny days, in two different years (2006 and 2007) and the sampling was done with entomological nets. Only single, solitary and mature females were used, and colour morph frequencies were estimated from the number of each morph divided by the total number of females.

## Authors' contributions

RSG designed the study, collected genetic samples, did laboratory work, analysed data and wrote the paper. MW designed the study, did laboratory work, analysed data and wrote the paper. ACR designed the study and wrote the paper. BH designed the study, analysed data and wrote the paper. All authors approved the final version of the manuscript.

## Supplementary Material

Additional file 1 **Bayesian assignment probabilities**. The average Bayesian assignment probabilities in each of the two genetic clusters (Q_1 _for *I. graellsii *(grey), and Q_2 _for *I. elegans *(grey dark)), for the nine Spanish populations of *I. elegans*. Click here for file
