# Supplementary figures and images for: Introgression and rapid species turnover in sympatric damselflies
Source: BMC Evol Biol. 2011 Jul 18;11:210. doi: 10.1186/1471-2148-11-210 (PMC3146444; doi:10.1186/1471-2148-11-210)

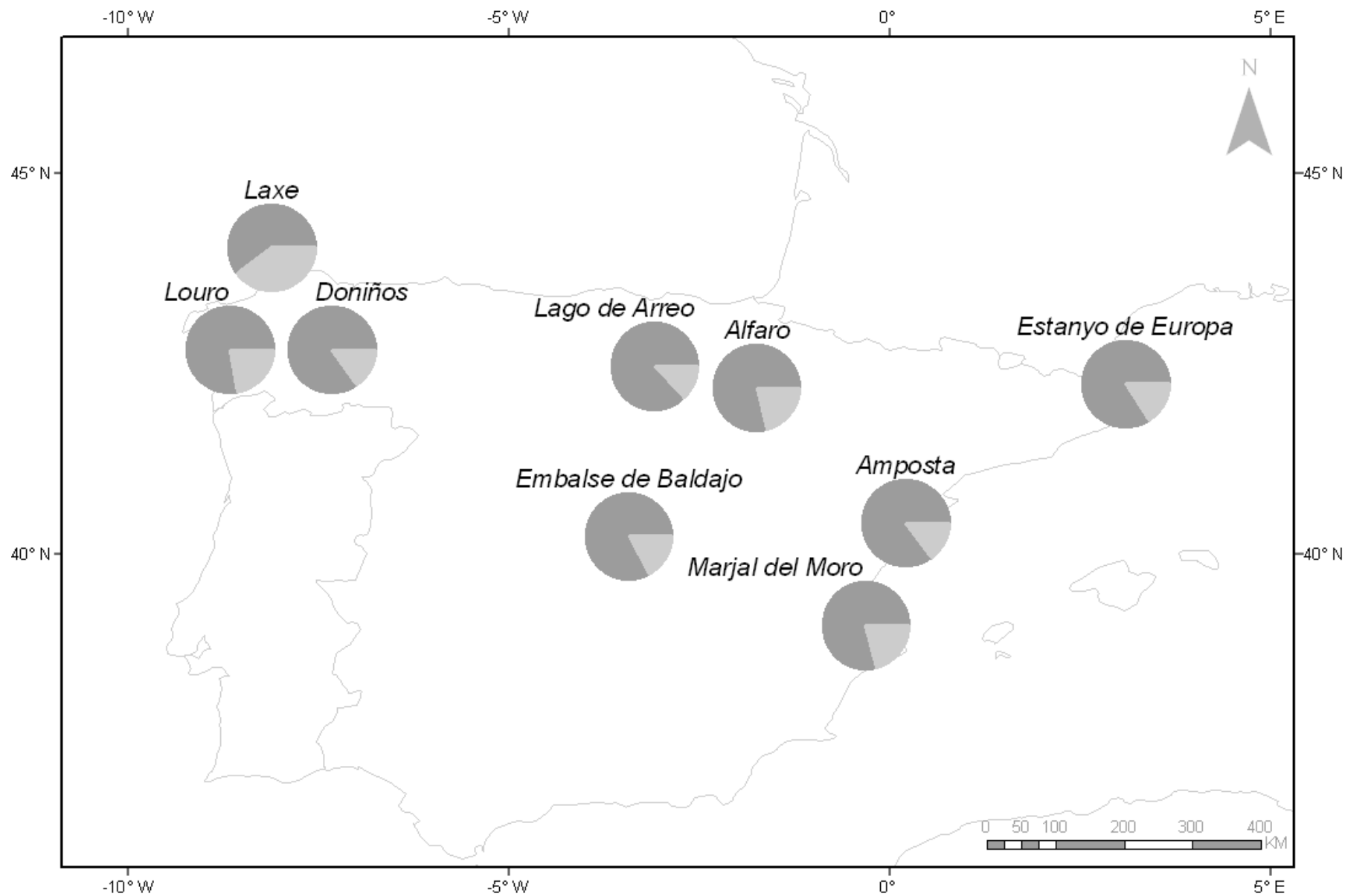

Supplement: Additional file 1 — Bayesian assignment probabilities. The average Bayesian assignment probabilities in each of the two genetic clusters (Q1 for I. graellsii (grey), and Q2 for I. elegans (grey dark)), for the nine Spanish populations of I. elegans. [file 1471-2148-11-210-S1.PDF]
